# Towards video-based injury risk assessment: predicting lifting loads from body pose trajectories

**DOI:** 10.1007/s00138-025-01758-w

**Published:** 2025-10-27

**Authors:** Zihao Zhu, Fangzhou Mu, Robert Radwin, Yin Li

**Affiliations:** 1 Department of Computer Sciences, University of Wisconsin-Madison, 1205 University Ave, Madison, WI 53706 USA; 2 Department of Biostatistics and Medical Informatics, University of Wisconsin-Madison, 610 Walnut St Room 201, Madison, WI 53726 USA; 3 Department of Industrial and Systems Engineering, University of Wisconsin-Madison, 1415 Engineering Drive, Madison, WI 53706 USA; 4 Department of Biomedical Engineering, University of Wisconsin-Madison, 1550 Engineering Drive, Madison, WI 53706 USA

**Keywords:** Video-based lifting load prediction, Ergonomics risk assessment, Human pose estimation

## Abstract

Manual material handling tasks, such as lifting and lowering, are ubiquitous across industry sectors. Overexertion during these tasks is among the leading causes of workplace injuries. Previous studies have shown that lifting load is a key factor in determining the risk of injury. However, existing methods for measuring the lifting load often rely on manual measurements, sensor fusion, or other techniques that are difficult to scale in practice. In this study, we present a vision-based approach to automatically predict lifting load by analyzing human body pose trajectories extracted from video alone. Specifically, our method employs person detection, visual tracking, and human body pose estimation to extract pose trajectories and their kinematic features, which are then used to train a Transformer model for load prediction. To evaluate our method, we conducted a human subjects study of 19 participants performing various lifting and lowering tasks with varying postures. Our method achieved an average accuracy of 74.8% to distinguish between light vs. heavy objects, and an average accuracy of 50.8% to identify three levels of lifting loads (light, medium, heavy) across lifting and lowering tasks. These results demonstrate a first step towards computer vision based solutions for automatic, noninvasive, scalable injury risk assessment for manual material handling tasks.

## Introduction

Manual material handling tasks are common in today’s manufacturing, logistics, and distribution sectors. These tasks, such as manual lifting or lowering, often involve repetitive motion and awkward postures, leading to a significant risk of musculoskeletal disorders (MSDs). MSDs are soft tissue injuries that can affect muscles, nerves, tendons, joints, and cartilage in the upper and lower limbs, neck and lower back. Overexertion during manual handling jobs ranked as the leading cause of disabling workplace injuries in the US from 2018 to 2024, costing businesses $12.49 billion in direct costs and accounted for over 21.5% among all causes of injury in 2024 [1]. To address this challenge, the Revised NIOSH Lifting Equation (RNLE) was developed in the early 1990 s [2] to assess the risk associated with lifting. The RNLE has since been widely adopted and provides a reliable tool for occupational health and safety risk analysis [3].

Conventionally, the application of the RNLE requires trained ergonomists to manually assess task conditions, either through direct field observations or by analyzing field survey videos [2]. The measurements include the horizontal and vertical distances between the lifted object and the body, the traveling distance of the object during lifting, etc. Based on these inputs, the RNLE calculates the recommended weight limit (RWL) for the lifting task. The lifting index (LI) is then computed as the ratio of the actual lifting load to the RWL, and the lifting load (i.e., the weight of the lifted object) is typically measured on-site. The LI serves as a risk indicator, providing a relative measure of the level of physical stress associated with a given lifting task.

Although the RNLE provides a robust assessment tool for risk analysis, its dependence on manual input from experts hinders its broader applicability. Several recent works [4–6] have considered using automated video analysis to derive measurements for RNLE from lifting videos. However, these approaches lack the critical capacity to estimate the lifting load of the object in the video. Recently, Zhou et al. [7] proposed to directly quantify LI from multiple lifting videos captured from different viewpoints, thus bypassing the need to estimate the lifting load. Specifically, their approach considers features including body posture, body motion, and facial expression, and employs convolutional neural networks and LSTMs to estimate LI. Despite encouraging results, the practicality of their method faces several major challenges. First, setting up multiple calibrated cameras that record a lifting task from varying viewpoints might not be feasible in the workspace. Second, there is insufficient evidence that LI is associated with facial expressions, and more importantly, facial expressions can be voluntarily altered, raising concerns about their reliability as risk indicators. Third, the processing of facial data may raise additional privacy and compliance challenges [8].

Beyond video-based methods, other sensing modalities such as surface electromyography (EMG) or instrumented load sensor could provide more direct estimates of exerted force. However, these approaches are invasive and often impractical in workplace settings. EMG requires electrodes attached to the skin and is most reliable for static exertions rather than dynamic lifts, while handheld or body-mounted device interfere with natural movement and reduce dexterity. For example, Marras et al. [9] developed a instrumented device measuring dynamic spinal load moment, but such systems are unsuitable for scalable deployment in real-word workplaces. By contrast, video-based analysis is entirely non-invasive, preserves natural behavior, and can be deployed at scale.

In this study, we investigate computer vision techniques to estimate lifting load in the RNLE from monocular video. Our core idea is that lifting load, as part of body kinetics (i.e., the forces acting on body segments), can be inferred from body kinematics (i.e., the motion of those segments), which can now be reliably extracted from video thanks to recent advances in computer vision [10].

Our idea is built on two key theoretical foundations. *First*, because body motion is governed by underlying forces according to biomechanical principles [11], it is theoretically feasible to solve the inverse problem: estimating the lifting load based on observed motion. However, this estimation is inherently ambiguous due to unknown factors such as individual body mass distribution and ground reaction forces, which can result in multiple plausible solutions for the same observed motion [12]. In such cases, incorporating prior knowledge about typical body motions and lifting loads can help reduce this ambiguity and improve estimation accuracy. *Second*, evidence from studies in intuitive physics shows that humans and animals are capable of inferring physical properties, such as object weight, based on visual observation alone [13]. Over decades, researchers have investigated how people perceive the weight of lifted objects, revealing that this ability is related to visual-haptic feedback, size-weight expectations, contextual information, and body motion [14–16]. Importantly, prior work has demonstrated that motion characteristics alone can be sufficient to support accurate weight estimation [17, 18].

**Fig. 1:**
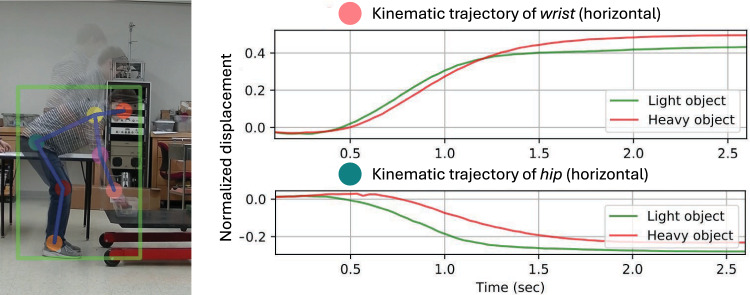
Body part trajectories under varying lifting loads. A comparison of hip and wrist kinematic trajectories during the lifting of heavy and light objects. Body part trajectories were automatically tracked in the video (*left*). Trajectories of wrist and hip were normalized and plotted (*right*)

To further validate our approach, we conducted a laboratory experiment in which multiple human subjects ($n=19$) performed two types of lifts — one with a light weight ($\le 5.4$ kg) and the other with a heavy weight ($\ge 10.9$ kg). Videos of the lifting were recorded, and key body joints (e.g., head, shoulders, elbows, wrists, hips, knees, and ankles) were tracked using a recent body pose estimation method [19]. Based on the tracking results, we plotted the average trajectories of the horizontal displacements of the hips and wrists over time, as shown in Fig. 1. These trajectories exhibit clear and consistent differences between the two lifting conditions. This empirical observation, combined with theoretical foundations, highlights the potential for new computational methods to exploit patterns in body part motion for predicting lifting loads relevant to the RNLE.

To this end, we present a computer vision approach that extracts trajectory patterns of key body parts from video and predicts the lifting load based on these patterns. Central to our method is a Transformer-based deep learning model [20] that learns to infer lifting loads from features derived from joint trajectories, which are obtained using an existing pose estimation method [19]. To evaluate our approach, we conducted a laboratory study involving 19 participants and a total of 1,022 lifting and lowering instances with varying postures. With leave-one-subject-out cross-validation, our approach achieved encouraging results in classifying different weight categories (e.g., light vs. heavy), significantly outperforming baseline methods using a 1D convolutional neural network (CNN) [21] or a long short-term memory (LSTM) network [22].

Our contributions are summarized as follows.

- We address a novel and practically important problem: estimating the weight of a lifted or lowered object from monocular video, designed for ergonomics risk assessment.
- We present a computer vision approach leveraging a Transformer-based deep learning model, which learns to analyze trajectories of key body parts extracted from video and predict the associated lifting load.
- We validate and demonstrate the effectiveness of our approach through a laboratory study involving 19 participants and over 1,000 lifting and lowering instances.

## Background and related work

### Biomechanics

Biomechanics [11] is a branch of science that studies the mechanics of living systems, such as the human body. One of the challenges in this field is to model the complex dynamics of the musculoskeletal system, using mathematical equations. A common approach is to use differential equations, which describe how a variable changes over time in relation to other variables. However, differential equations also pose some difficulties for modeling human body motion, such as the high dimensionality, the sensitivity to initial conditions, and the numerical instability. To simplify musculoskeletal system and improve estimation of kinetics, an articulated rigid body system is often considered.

To connect kinematics and kinetics during lifting, D’Alembert’s principle is applied, in which the body system remain equilibrium under real force and inertial force. In this case, an articulated rigid body system is assumed, where the body shape remains unchanged and the body pose is uniquely defined using a collection of joints (e.g., 23 in SMPL [23]). With stationary feet on the floor, it is safe to assume no global translation or rotation of the human body. Specifically, denote $\textbf{q}(t)\in \mathbb {R}^d$ as the generalized coordinates encapsulating all joints’ degree of freedom (DOF) at time step *t* (e.g., all joints’ SMPL rotation angles at *t*). Let $\dot{\textbf{q}}(t)$ and $\ddot{\textbf{q}}(t)$ be the generalized velocity and acceleration of $\textbf{q}$ at *t*. The body dynamics are governed by the Euler-Lagrange equation

1 $$\begin{aligned} M(\textbf{q}(t))\ddot{\textbf{q}}(t) + C(\textbf{q}(t),\dot{\textbf{q}}(t)) = \textbf{G}(t), \end{aligned}$$

where $M(\textbf{q}(t))\in \mathbb {R}^{d\times d}$ is the mass matrix determined by $\textbf{q}(t)$ and physical parameters including the body mass distribution and inertia. $C(\textbf{q}(t),\dot{\textbf{q}}(t))\in \mathbb {R}^d$ represents the generalized bias forces, including Coriolis, centrifugal, and gravitational forces and torques. $\textbf{G}(t)\in \mathbb {R}^d$ is the generalized forces (i.e., kinetics), including both external forces (e.g., lifting load and ground reaction force) and internal forces (e.g., joint actuation).

Eq. 1 establishes the relationship between body kinematics ($\textbf{q}$, $\dot{\textbf{q}}$, and $\ddot{\textbf{q}}$) and kinetics (*G*). Notably, if joint DOFs $\textbf{q}$ can be precisely tracked over time, and assuming mass matrix *M* and generalized bias forces *C* are known, the generalized forces *G* including the lifting load can be computed, although separating the external vs. internal forces (e.g., lifting load vs. wrist actuation) still remains challenging. Nevertheless, this biomechanical analysis highlights the feasibility of our work — to estimate lifting loads based on body joint trajectories that can be readily tracked from monocular video using computer vision.

### Intuitive physics from video

Estimating lifting load from video is closely related to intuitive physics — the innate ability of humans and animals for reasoning about physical properties without formal education or training. Convincing evidence suggests that this cognitive process differs fundamentally from Newtonian physics [24, 25]. Computational approaches for intuitive physics often build on Bayesian inference [26, 27], incorporating uncertainty in sensory input, probabilistic simulations with a mental physics engine, and prior knowledge about physical attributes and events [13, 28–31].

Recently, deep learning models have been explored to predict physical properties directly from visual inputs [32, 33]. Notably, several studies have focused on estimating forces from monocular video. These works demonstrated that contact forces exerted by hands or body parts during their interaction with objects can be predicted using deep neural networks based on motion trajectories, and further optimized using biomechanical analysis [32, 34]. The neural networks can be trained with supervisions from ground-truth contact forces measured by additional sensors [34], by comparing predicted trajectories against those from a physics simulation engine [33], or by observing the deformation of interacted objects [35]. Building on this line of work, our approach aims to estimate lifting load from monocular video. However, unlike prior methods, our work is specifically designed for ergonomic risk assessment with the RNLE, making it directly applicable to occupational safety and health.

### Video-based ergonomic risk assessment

The Revised NIOSH Lifting Equation (RNLE) [2] typically relies on expert observation and manual measurement of body posture and motion to assess the risk of musculoskeletal injury. To automate this process, several prior work has explored computer vision methods for analyzing task videos [36–39]. A common workflow in video-based ergonomics risk analysis follows a two-stage framework. In the first stage, body posture and motion are extracted using vision algorithms to derive necessary parameters. In the second stage, these parameters are input into a chosen ergonomic assessment tool to compute the corresponding risk level. A few recent studies have explored end-to-end learning approaches for ergonomic assessment [6, 7], where models directly predict risk scores from input video without computing intermediate parameters. While our method adopts an end-to-end learning framework for estimating lifting load from video, it is designed to be compatible with existing ergonomics risk assessment tools (e.g., the RNLE) and can be directly integrated into their workflows.

**Fig. 2:**
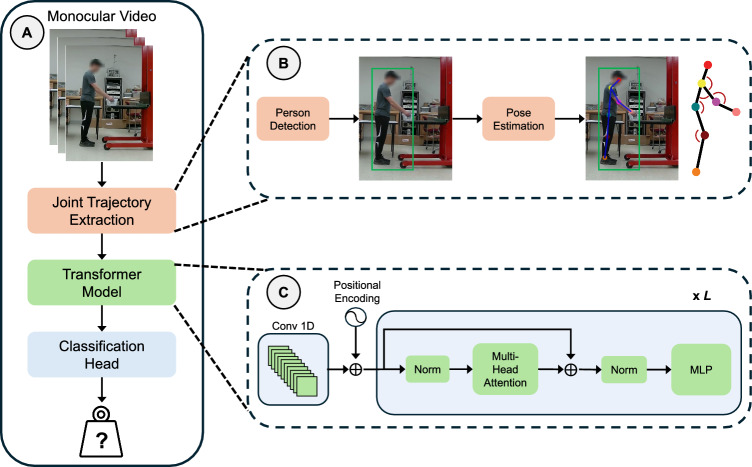
*Method overview*. **A** Our method extracts trajectory features of human body joints from monocular video, processes them using a Transformer model, and predicts the associated lifting load. **B** The trajectory features are obtained by integrating person detection, pose estimation, and visual tracking, and include joint positions, joint angles, as well as joint velocities and accelerations. **C** A Transformer model is employed to process the time series of trajectory features, capturing temporal dependencies relevant to lifting load estimation

## Proposed method

*Scope and problem formulation*. Our goal is to automatically quantify the lifting load, defined as the weight of the lifted object, from an input monocular video. As an initial step, we consider videos captured by a static camera containing a single worker performing one lift, and focus on predicting the categories of lifting load (e.g., light vs. medium vs. heavy). Further, we assume that the camera is facing the sagittal plane of lifting and thus captures the side views of the lifts. Although this setting is constrained, it is well suited for our application scenarios; industrial practitioners can control the capturing angle and record multiple videos of a single lift, in order to employ ergonomic assessment tools. When multiple workers are presented (e.g., in a collaborative task), visual object tracking [40] can be used to identify individual workers before the application of our method.

*Method overview*. To this end, we propose a novel framework for predicting the lifting load based on body posture and motion tracked from the input video. Specifically, our approach, as illustrated in Fig. 2A, consists of two stages. In the first stage, kinematic trajectories of 2D key joints positions and joint angles, as well as their velocities and accelerations, are extracted from videos based on computer vision algorithms. In the second stage, extracted trajectories are processed by a Transformer network [20] to model long-range dependencies and to predict the lifting load. In what follows we describe details of our approach.

### Kinematic features extraction from video

To extract kinematic trajectories of key body parts from monocular video, we integrate well-established computer vision techniques following a top-down approach. Specifically, we employ person detection [41] to identify the individual performing the lift, body pose estimation [19] to detect key joints of the identified individual, and visual tracking [19] to associate these detected joints across video frames. Our pipeline outputs the trajectories of 17 body joints in the 2D image plane, following the standard format in [42].

Since we assume that each lifting is captured from the sagittal plane, only one side (left or right) of the body is visible in the input video, and the other side is assumed to be approximately symmetric. Accordingly, we select seven key joints sufficient to characterize the lifting motion, and retain only their trajectories for further analysis. These selected joints include the head, and the right (or left) shoulder, elbow, wrist, hip, knee, and ankle, as illustrated in Fig. 2B. To account for variations in height and camera distance, we further normalize the 2D coordinates within the trajectories. Specifically, at each frame, joint positions are re-centered with respect to the center of the detected human bounding box, and further normalized by the maximum bounding box height observed across the video.

With seven key body parts extracted, we then compute additional features that enhance the model’s capacity to interpret the underlying dynamics of the subject. The features include joint angles, joint velocity, and joint acceleration. Notably, these features are derived from the biomechanical analysis in Eq. 1, corresponding to the generalized coordinates, as well as their velocity and acceleration. Specifically, we consider 5 joint angles in the sagittal plane, calculated from 7 tracked joints. Each angle was computed using two adjacent limb segments, derived from 2D joints (see Fig. 2B right most):

- Neck-shoulder to shoulder-elbow
- Shoulder-elbow to elbow-wrist
- Shoulder-elbow to shoulder-hip
- Shoulder-hip to hip-knee
- Hip-knee to knee-ankle

Further, velocity is computed as the rate of change of body parts position over time, and acceleration is calculated as the rate of change of body parts’ velocity over time.

Formally, a trajectory spanning *T* video frames (i.e., time steps) is represented as a time series $\textbf{X}=\{\textbf{x}_1, \textbf{x}_2, \ldots , \textbf{x}_T\}$. $\textbf{x}_t$ denotes a 47 dimensional feature vector at frame *t*, consisting of

- normalized 2D joint positions (7 joints) $\textbf{r}$;
- 2D joint angles (5 angles) $\textbf{ang}$;
- 2D joint velocity (7 joints) $\textbf{v}$;
- 2D joint acceleration (7 joints) $\textbf{a}$.

A key design choice of our method is to only consider features derived from joint positions, without directly using raw pixel values in the video. This design choice helps mitigate potential biases arising from variations in body shape, clothing, lighting condition, or background clutter.

### Lifting load prediction with transformer

We design a Transformer-based deep neural network to predict the lifting load *Y* based on the input trajectory features $\textbf{X}$. Our model consists of (1) a *projection* module that embeds $\textbf{X}$ into a latent space; (2) a series of *Transformer* blocks that processes the embedded features; and (3) a *classification* head that predicts the lifting load *Y* based on the processed features.

*Projection*. Our projection $\textbf{E}$ is a shallow convolutional network with ReLU as the activation function, defined as

2 $$\begin{aligned} \textbf{Z}^0 = [ \textbf{E}(\textbf{x}_1), \textbf{E}(\textbf{x}_2), \ldots , \textbf{E}(\textbf{x}_T) ]^T + \textbf{E}_{pos}, \end{aligned}$$

where $\textbf{E}(\textbf{x}_i) \in \mathbb {R}^{D}$ is the embedded feature of $\textbf{x}_i$ with *D* as the embedding dimension. $\textbf{E}_{pos} \in \mathbb {R}^{T \times D}$ is the position embedding as used in [20], and is added to the embedded features.

*Transformer*. Multiple Transformer blocks with self-attention [20] further process $\textbf{Z}^0$. To make the paper self-contained, we briefly introduce self-attention and Transformer. Concretely, self-attention computes a weighted average of features with the weight proportional to a similarity score between pairs of input features. Given $\textbf{Z}^0 \in \mathbb {R}^{T \times D}$ with *T* time steps of *D* dimensional features, $\textbf{Z}^0$ is projected using $\textbf{W}_Q \in \mathbb {R}^{D \times D_q}$, $\textbf{W}_K \in \mathbb {R}^{D \times D_k}$, and $\textbf{W}_V \in \mathbb {R}^{D\times D_v}$ to extract feature representations $\textbf{Q}$, $\textbf{K}$, and $\textbf{V}$, referred to as query, key and value respectively with $D_k = D_q$. The outputs $\textbf{Q}$, $\textbf{K}$, $\textbf{V}$ are computed as

3 $$\begin{aligned} \textbf{Q} = \mathbf {\textbf{Z}^0} \textbf{W}_Q, \quad \textbf{K} = \mathbf {\textbf{Z}^0} \textbf{W}_K, \quad \textbf{V} = \mathbf {\textbf{Z}^0} \textbf{W}_V. \end{aligned}$$

The output of self-attention is given by

4 $$\begin{aligned} \textbf{S} = \text {softmax}\left( \textbf{Q} \textbf{K}^T / \sqrt{D_q} \right) \textbf{V}, \end{aligned}$$

where $\textbf{S}\in \mathbb {R}^{T \times D}$ and $\text {softmax}$ is performed *row-wise*. A multiheaded self-attention ($\operatorname {MSA}$) further adds several self-attention operations in parallel.

A Transformer block further combines the MSA, layer norm ($\operatorname {LN}$), a multi-layer perceptron ($\operatorname {MLP}$), and residual connections [20]. A Transformer network is formed by stacking *L* blocks together, and processes the input in a sequential manner. This is given by

5 $$\begin{aligned} \begin{aligned} {\bar{\textbf{Z}}}^\ell&= \operatorname {MSA}(\operatorname {LN}(\textbf{Z}^{\ell -1})) + \textbf{Z}^{\ell -1}, \\ {\hat{\textbf{Z}}}^\ell&= \operatorname {MLP}(\operatorname {LN}({\bar{\textbf{Z}}}^{\ell })) + {\bar{\textbf{Z}}}^{\ell }, \end{aligned} \end{aligned}$$

where $l \in [1,..., L]$ indexes the blocks. For the *l*-th block, ${\bar{\textbf{Z}}}^\ell$ denotes the intermediate features and ${\hat{\textbf{Z}}}^\ell$ the output.

*Classification*. Finally, processed features ${\hat{\textbf{Z}}}^L$ from the last Transformer block is average pooled across the temporal dimension, and fed into an MLP for the final classification. The average pooling yields a single vector representing the trajectory. Based on this vector representation, an MLP further predicts the probability of discrete load categories (i.e., *P*(*Y*)), corresponding to various lifting loads (e.g., light vs. medium vs. heavy).

### Model training and inference

- *Training loss.* Our model is trained using supervised learning with ground-truth lifting loads as labels. We use cross-entropy loss with a Softmax activation for output normalization. Notably, the target categories represent different lifting loads, which are inherently ordered.
- *Data augmentation.* To improve the robustness of the model, we apply data augmentation during training. Specifically, we randomly flip the horizontal positions of all joints to simulate lifting motions captured from either side of the body in the sagittal plane. Additionally, we add small Gaussian noise to all trajectory features to account for variability and noise in the feature extraction process.
- *Inference.* To further improve model performance, we adopt test-time data augmentation. Specifically, both the original joint trajectory and its horizontally mirrored version are fed into the model, and the final prediction is obtained by averaging their outputs.

## Experiments and results

### Experiment design and dataset

To evaluate the performance of our method, we use data collected in a human subject study from our prior work [43]. The study, which was conducted in a laboratory setting and approved by the UW-Madison IRB, recruited 19 participants from local residents, including 6 female and 13 male adults with average age $22.8\pm 7.8$ (min: 18 and max: 53). All participants consented to participated in the study.

**Fig. 3:**
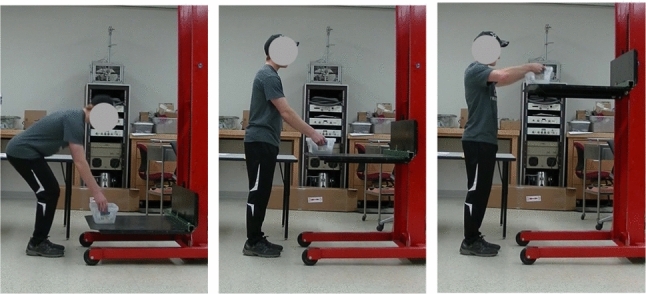
Lifting and lowering postures. Each participant performed multiple lifting and lower tasks at three postures with varying heights (from left to right): *mid-shin*, *knuckle*, and *shoulder*

*Study design.* Prior to each session, an experimenter first introduced the protocol and answered any relevant questions from the participants. During the session, the participants were asked to perform lifting and lowering tasks at three different postures with vertical heights around the level of the participant’s mid-shin, knuckle, and shoulder heights, as shown in Fig. 3. For each posture, a maximum of 9 symmetric lifting and lowering tasks of varying loads weighing were performed, where the loads were randomly selected from the set [2.7, 4.1, 5.4, 6.8, 8.2, 9.5, 10.9, 12.2, 13.6] kg, and the horizontal distance was controlled within 30 cm. The loads were positioned in the same opaque box, such that no visual cues can be used to gauge their weight from the video.

*Data recording and processing.* Lifting videos were captured by a camera facing the sagittal plane of lifting at a resolution of 1920$\times$1080 with a frame rate of 30 Hz. Loads were recorded for each lifting or lowering instance. Onsets and offsets of each lifting or lowering instance were manually annotated. Instances without load recording or blocked by the experimenter were excluded. For each instance, trajectories features, as described in Sect. 3.1, were extracted from the video. A total of 1022 lifting and lowering instances and spanning over 2 h of video recordings were retained and considered in our experiments.

**Table 1:** *Main results*: accuracy of our method to quantify lifting loads and comparison with baselines

Method	Binary accuracy (%)		Three-way accuracy (%)	
	Lowering	Lifting	Lowering	Lifting
CNN	61.93 ± 11.02	70.95 ± 10.49	42.81 ± 8.75	45.60 ± 6.57
LSTM	65.76 ± 10.39	69.35 ± 7.46	44.25 ± 7.95	47.00 ± 6.67
Transformer (ours)	72.65 ± 11.76	76.86 ± 10.22	49.40 ± 7.43	52.16 ± 9.50

**Fig. 4:**
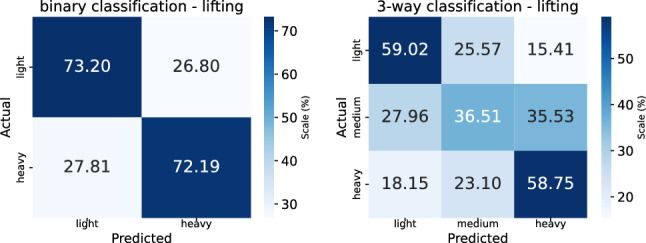
Confusion matrices for binary (*left*) and three-way (*right*) prediction of lifting load

### Implementation details

*Model architecture.* Our model was implemented using PyTorch [44]. The projection step included two convolution layers with ReLU. The main model consisted of two Transformer blocks (each with four attention heads), followed by an average pooling and then a linear classification head. The dimension of all intermediate embeddings and features was set to 188 (4 times the input feature dimension).

*Training details.* The model was trained with 100 epochs including 10 warm up epochs, using a single NVIDIA A6000 GPU. To accommodate varying input length, masking was implemented and feature pooling was restricted to valid slots. To prevent overfitting, we incorporated dropout operations in all Transformer blocks with a rate of 0.1, including attention dropout, projection dropout, and path dropout. We opted for the AdamW optimizer with an initial learning rate of 0.001, complemented with a linear warm-up and cosine annealing schedule. This training scheme was used for our method and all baselines.

### Evaluation protocol, metrics and baselines

*Protocol and metrics.* The lifting loads are categorized into three levels of exertion: light ($2.7-5.4$ kg), medium ($6.8-9.5$ kg), and heavy ($10.9-13.6$ kg). Classification performance averaged across all postures are evaluated in two settings: (1) binary classification between light and heavy loads, and (2) three-way classification among all three categories. To account for the limited dataset size and ensure robustness, we adopt a leave-one-subject-out cross-validation strategy. Specifically, the experiments are repeated *N* times, where *N* is the number of participants. In each fold, data from one participant is used for testing, while the remaining participants’ data are used for training. Classification accuracy for lifting and lowering and its standard deviation, aggregated across all folds, are reported as the final performance metrics.

**Fig. 5:**
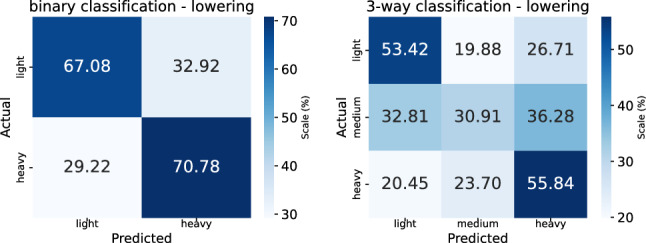
Confusion matrices for binary (*left*) and three-way (*right*) prediction of lowering load

*Baseline methods.* Two representative baselines are considered in our experiments, including (1) a 1D Convolutional Neural Network (CNN); and (2) a recurrent neural network using a Long Short-Term Memory network (LSTM). Both models have been proven effective for time series analysis. The CNN is instantiated using a 1D residual network [45] including depthwise convolutions for projection, 2 residual blocks for processing, and an average pooling with linear head for classification. The LSTM uses convolutions for projection and includes 2 bi-directional LSTM layers, followed by a linear head attached to the last hidden state for classification. The design of our baselines is informed by their empirical results.

**Fig. 6:**
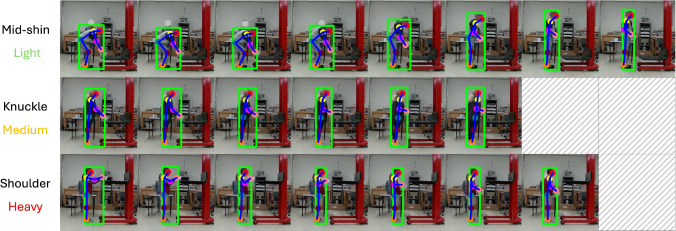
*Results visualization*. Frames from input videos and their corresponding pose tracking results are shown. For visualization purposes, shorter sequences are padded with gray frames. From *top to bottom*, each row represents a correctly classified example from a different participant, spanning all lifting postures (mid-shin, knuckle, and shoulder) and load levels (light, medium, and heavy)

### Results and analysis

*Comparison to baselines.* Our main results for predicting lifting and lowering loads are summarized in Table 1. Our method significantly outperforms the baselines. Specifically, the baseline CNN achieves accuracies of $61.93\%/70.95\%$ (binary) and $42.81\%/45.60\%$ (three-way) for lowering and lifting, respectively. LSTM, enhanced with improved temporal molding capabilities, marginally outperforms CNN, showing accuracies of $65.76\%/69.35\%$ (binary) and $44.25\%/47.00\%$ (three-way) for lowering and lifting, respectively. Importantly, our Transformer model attains accuracies of $72.65\%$/$76.86\%$ (binary) and $49.40\%$/$52.16\%$ (three-way) for lowering and lifting. Across all cases, our method outperforms the best performing baseline by 5.2-7.5% in accuracy. Further, a McNemar’s test [46] shows that the results of our Transformer model is significantly better than the CNN ($p<0.001$) and LSTM ($p<0.001$) baselines.

Two additional observations emerge from the results. First, the accuracy of three-way classification is consistently lower than that of binary classification, reflecting the increased complexity of the task. Second, across all models, classification accuracy is significantly higher for lifting tasks compared to lowering tasks, suggesting that lifting motions may carry more discriminative cues for inferring load levels.

**Fig. 7:**
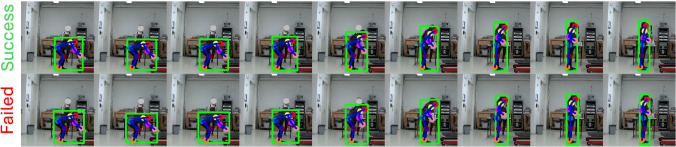
*Successful and failure cases for lifting load prediction*. *Top*: The ground-truth lifting load is light (4.1 kg), and the model correctly predicts it as light. *Bottom*: The ground-truth lifting load is light (5.4 kg), but the model incorrectly predicts it as heavy

**Fig. 8:**
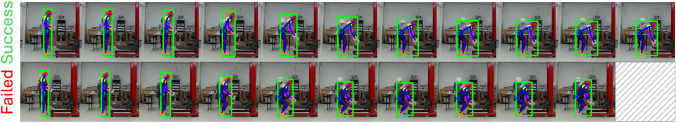
*Successful and failure cases for lowering load prediction*. *Top*: The ground-truth lowering load is medium (8.2 kg), and the model correctly predicts it as medium. *Bottom*: The ground-truth lowering load is medium (9.5 kg), but the model incorrectly predicts it as light

**Table 2:** Ablation on model design: Effects of number of Transformer blocks *L* in our model

Method	Binary accuracy (%)		Three-way accuracy (%)	
	Lowering	Lifting	Lowering	Lifting
Ours ($L=2$)	72.65 ± 11.76	76.86 ± 10.22	49.40 ± 7.43	52.16 ± 9.50
Ours ($L=4$)	71.52 ± 12.33	76.35 ± 9.49	49.85 ± 7.93	53.40 ± 11.38
Ours ($L=6$)	71.76 ± 11.35	77.17 ± 9.97	53.17 ± 11.12	52.24 ± 8.93

**Table 3:** Model generalization: intra-subject versus inter-subject performance

Method	Binary accuracy (%)		Three-way accuracy (%)	
	Lowering	Lifting	Lowering	Lifting
Inter-subjects	72.65 ± 11.76	76.86 ± 10.22	49.40 ± 7.43	52.16 ± 9.50
Intra-subjects	72.14 ± 3.32	78.24 ± 4.21	51.75 ± 1.33	55.04 ± 2.00

*Results analysis*. Confusion matrices, corresponding to the results in Table 1, are shown in Fig. 4 (for lifting) and in Fig. 5 (for lowering). The plots reveal a consistent challenge in identifying medium-weight loads during three-way classification. Specifically, our model correctly predicts the medium load in only 36.51% of lifting cases and 30.91% of lowering cases. This suggests that medium-weight lifting dynamics may lack sufficiently distinctive motion patterns compared to light or heavy loads, making it more difficult for the model to learn discriminative features.

Moreover, sample results of our method in tandem with the video features, are visualized in Figs. 6, 7, and 8. Figure 6 demonstrates sample results across different postures and load levels. Figures 7 and 8 show successful and failure cases for three-way classification of lifting and lowering loads, respectively. Despite accurate body pose tracking, our model is still prone to misclassifications at times.

Additionally, a breakdown of the results across postures is included in the last rows of each blocks in Tables 4, 5, 6, and 7. Importantly, across both binary and three-way classification settings, and for all three postures in lifting and lowering, our method outperforms the best performing baseline in the majority of cases (11 out of 12).

**Table 4:** Detailed results for binary lifting load prediction

Method	Features	Shoulder	Knuckle	Mid-shin	All
CNN	$\textbf{r}$	$65.05 \pm 21.33$	$64.07 \pm 16.94$	$75.93 \pm 16.87$	$67.91 \pm 10.49$
	$\textbf{r}+\textbf{v}$	$72.69 \pm 21.30$	$64.26 \pm 13.84$	$75.74 \pm 18.47$	$70.12 \pm 11.58$
	$\textbf{r}+\textbf{a}$	$64.73 \pm 20.44$	$63.52 \pm 10.04$	$75.00 \pm 17.21$	$66.99 \pm 9.70$
	$\textbf{r}+\textbf{ang}$	$69.77 \pm 14.49$	$65.83 \pm 16.13$	$73.24 \pm 19.76$	$68.93 \pm 9.62$
	$\textbf{r}+\textbf{v}+\textbf{a}$	$71.85 \pm 19.90$	$67.41 \pm 15.44$	$75.74 \pm 18.13$	$71.38 \pm 10.23$
	$\textbf{r}+\textbf{v}+\textbf{ang}$	$69.17 \pm 16.62$	$70.65 \pm 18.34$	$76.30 \pm 17.41$	$\mathbf {71.62 \pm 9.26}$
	$\textbf{r}+\textbf{a}+\textbf{ang}$	$74.81 \pm 17.27$	$62.13 \pm 13.49$	$72.59 \pm 20.32$	$69.19 \pm 8.49$
	$\textbf{r}+\textbf{v}+\textbf{a}+\textbf{ang}$	$73.94 \pm 16.42$	$68.33 \pm 17.85$	$73.24 \pm 18.13$	$70.95 \pm 10.49$
LSTM	$\textbf{r}$	$73.01 \pm 15.24$	$70.28 \pm 16.10$	$65.74 \pm 16.41$	$69.05 \pm 9.84$
	$\textbf{r}+\textbf{v}$	$71.34 \pm 14.28$	$68.43 \pm 17.13$	$63.89 \pm 10.70$	$67.45 \pm 7.85$
	$\textbf{r}+\textbf{a}$	$71.30 \pm 21.96$	$70.42 \pm 15.34$	$64.17 \pm 14.31$	$68.75 \pm 9.15$
	$\textbf{r}+\textbf{ang}$	$68.10 \pm 16.78$	$71.99 \pm 16.44$	$67.96 \pm 11.31$	$68.61 \pm 7.73$
	$\textbf{r}+\textbf{v}+\textbf{a}$	$73.70 \pm 17.26$	$71.80 \pm 16.82$	$68.61 \pm 9.85$	$71.36 \pm 8.72$
	$\textbf{r}+\textbf{v}+\textbf{ang}$	$72.50 \pm 16.64$	$73.61 \pm 15.42$	$69.26 \pm 15.79$	$70.83 \pm 8.10$
	$\textbf{r}+\textbf{a}+\textbf{ang}$	$74.72 \pm 15.85$	$70.93 \pm 15.42$	$72.13 \pm 14.84$	$\mathbf {71.64 \pm 7.57}$
	$\textbf{r}+\textbf{v}+\textbf{a}+\textbf{ang}$	$74.49 \pm 16.89$	$71.94 \pm 15.04$	$65.56 \pm 13.53$	$69.35 \pm 7.46$
Transformer (ours)	$\textbf{r}$	$79.91 \pm 23.29$	$81.71 \pm 18.49$	$73.33 \pm 15.85$	$77.80 \pm 10.73$
	$\textbf{r}+\textbf{v}$	$78.89 \pm 20.18$	$78.44 \pm 22.81$	$75.06 \pm 16.18$	$78.21 \pm 8.90$
	$\textbf{r}+\textbf{a}$	$88.15 \pm 19.59$	$72.22 \pm 26.56$	$78.18 \pm 16.18$	$\mathbf {79.98 \pm 11.30}$
	$\textbf{r}+\textbf{ang}$	$84.91 \pm 13.42$	$76.20 \pm 23.30$	$73.73 \pm 21.73$	$77.46 \pm 11.21$
	$\textbf{r}+\textbf{v}+\textbf{a}$	$86.85 \pm 17.36$	$81.00 \pm 19.20$	$75.39 \pm 15.33$	$79.46 \pm 9.99$
	$\textbf{r}+\textbf{v}+\textbf{ang}$	$84.77 \pm 15.88$	$72.96 \pm 16.50$	$70.15 \pm 16.14$	$74.98 \pm 7.95$
	$\textbf{r}+\textbf{a}+\textbf{ang}$	$84.17 \pm 20.66$	$72.28 \pm 22.27$	$69.12 \pm 19.12$	$74.30 \pm 8.68$
	$\textbf{r}+\textbf{v}+\textbf{a}+\textbf{ang}$	$87.31 \pm 16.16$	$76.05 \pm 24.80$	$69.48 \pm 15.12$	$76.86 \pm 10.22$

Bold values indicate the best overall accuracy among each method

**Table 5:** Detailed results for three-way lifting load prediction

Model	Features	Shoulder	Knuckle	Mid-shin	All
Conv	$\textbf{r}$	$45.55 \pm 17.68$	$42.23 \pm 10.14$	$49.94 \pm 13.80$	$45.44 \pm 9.55$
	$\textbf{r}+\textbf{v}$	$46.51 \pm 17.82$	$44.13 \pm 15.97$	$52.24 \pm 14.88$	$46.66 \pm 7.31$
	$\textbf{r}+\textbf{a}$	$42.67 \pm 19.30$	$39.47 \pm 13.48$	$46.83 \pm 12.77$	$42.47 \pm 8.82$
	$\textbf{r}+\textbf{ang}$	$46.28 \pm 18.04$	$42.19 \pm 12.51$	$49.73 \pm 15.77$	$45.11 \pm 6.59$
	$\textbf{r}+\textbf{v}+\textbf{a}$	$45.77 \pm 16.05$	$41.40 \pm 8.02$	$55.35 \pm 13.72$	$47.03 \pm 6.73$
	$\textbf{r}+\textbf{v}+\textbf{ang}$	$52.17 \pm 20.39$	$43.65 \pm 11.40$	$51.81 \pm 13.22$	$\mathbf {48.09 \pm 8.34}$
	$\textbf{r}+\textbf{a}+\textbf{ang}$	$49.57 \pm 18.61$	$40.02 \pm 13.15$	$50.42 \pm 16.45$	$45.32 \pm 7.73$
	$\textbf{r}+\textbf{v}+\textbf{a}+\textbf{ang}$	$46.96 \pm 13.94$	$40.90 \pm 10.86$	$49.40 \pm 13.14$	$45.60 \pm 6.57$
LSTM	$\textbf{r}$	$50.68 \pm 11.87$	$50.02 \pm 14.60$	$46.20 \pm 10.50$	$\mathbf {48.78 \pm 8.50}$
	$\textbf{r}+\textbf{v}$	$47.45 \pm 17.35$	$46.31 \pm 14.88$	$46.03 \pm 14.35$	$47.29 \pm 9.23$
	$\textbf{r}+\textbf{a}$	$45.00 \pm 16.96$	$47.89 \pm 14.30$	$44.44 \pm 9.95$	$46.11 \pm 7.78$
	$\textbf{r}+\textbf{ang}$	$48.70 \pm 11.18$	$47.33 \pm 12.55$	$45.39 \pm 8.93$	$46.81 \pm 5.99$
	$\textbf{r}+\textbf{v}+\textbf{a}$	$46.19 \pm 11.15$	$46.35 \pm 12.71$	$45.46 \pm 10.16$	$45.91 \pm 7.40$
	$\textbf{r}+\textbf{v}+\textbf{ang}$	$52.52 \pm 12.28$	$47.26 \pm 11.95$	$45.78 \pm 9.78$	$47.90 \pm 7.02$
	$\textbf{r}+\textbf{a}+\textbf{ang}$	$47.58 \pm 12.21$	$49.04 \pm 10.71$	$44.57 \pm 11.73$	$47.01 \pm 6.32$
	$\textbf{r}+\textbf{v}+\textbf{a}+\textbf{ang}$	$45.68 \pm 11.50$	$49.89 \pm 14.82$	$44.61 \pm 10.42$	$47.00 \pm 6.67$
Transformer (ours)	$\textbf{r}$	$54.61 \pm 19.75$	$45.82 \pm 14.67$	$47.65 \pm 13.01$	$49.25 \pm 9.01$
	$\textbf{r}+\textbf{v}$	$57.00 \pm 24.67$	$50.60 \pm 16.18$	$49.77 \pm 16.79$	$52.95 \pm 10.59$
	$\textbf{r}+\textbf{a}$	$53.83 \pm 22.71$	$49.02 \pm 15.68$	$58.17 \pm 20.09$	$52.74 \pm 10.15$
	$\textbf{r}+\textbf{ang}$	$60.38 \pm 16.98$	$48.75 \pm 16.63$	$48.95 \pm 15.67$	$51.91 \pm 9.23$
	$\textbf{r}+\textbf{v}+\textbf{a}$	$56.77 \pm 22.47$	$54.38 \pm 17.14$	$51.28 \pm 13.92$	$\mathbf {53.92 \pm 11.09}$
	$\textbf{r}+\textbf{v}+\textbf{ang}$	$57.96 \pm 19.76$	$48.07 \pm 16.57$	$52.22 \pm 17.67$	$53.26 \pm 8.99$
	$\textbf{r}+\textbf{a}+\textbf{ang}$	$56.81 \pm 18.47$	$44.10 \pm 15.36$	$54.92 \pm 16.62$	$52.19 \pm 9.35$
	$\textbf{r}+\textbf{v}+\textbf{a}+\textbf{ang}$	$56.08 \pm 23.96$	$45.14 \pm 16.80$	$56.78 \pm 18.02$	$52.16 \pm 9.50$

Bold values indicate the best overall accuracy among each method

**Table 6:** Detailed results for binary lowering load prediction

Method	Features	Shoulder	Knuckle	Mid-shin	All
CNN	$\textbf{r}$	$64.58 \pm 22.38$	$68.87 \pm 14.46$	$62.31 \pm 12.74$	$65.11 \pm 9.17$
	$\textbf{r}+\textbf{v}$	$64.81 \pm 24.48$	$66.05 \pm 15.53$	$64.54 \pm 12.68$	$64.92 \pm 12.48$
	$\textbf{r}+\textbf{a}$	$60.79 \pm 19.72$	$64.72 \pm 9.53$	$66.11 \pm 13.39$	$63.64 \pm 9.10$
	$\textbf{r}+\textbf{ang}$	$64.26 \pm 15.96$	$66.83 \pm 15.41$	$62.87 \pm 17.17$	$64.34 \pm 12.93$
	$\textbf{r}+\textbf{v}+\textbf{a}$	$63.24 \pm 20.07$	$72.01 \pm 13.78$	$65.28 \pm 12.88$	$\mathbf {66.62 \pm 9.90}$
	$\textbf{r}+\textbf{v}+\textbf{ang}$	$60.09 \pm 17.05$	$67.36 \pm 18.08$	$61.57 \pm 12.96$	$63.21 \pm 11.78$
	$\textbf{r}+\textbf{a}+\textbf{ang}$	$58.61 \pm 15.57$	$66.27 \pm 15.09$	$56.94 \pm 13.11$	$60.72 \pm 9.85$
	$\textbf{r}+\textbf{v}+\textbf{a}+\textbf{ang}$	$58.43 \pm 16.20$	$69.10 \pm 19.22$	$56.85 \pm 10.97$	$61.93 \pm 11.02$
LSTM	$\textbf{r}$	$70.14 \pm 18.25$	$68.05 \pm 15.01$	$66.20 \pm 18.10$	$\mathbf {68.33 \pm 12.39}$
	$\textbf{r}+\textbf{v}$	$67.13 \pm 17.07$	$68.22 \pm 14.46$	$62.50 \pm 16.26$	$66.02 \pm 9.67$
	$\textbf{r}+\textbf{a}$	$60.39 \pm 23.29$	$65.07 \pm 13.83$	$62.59 \pm 18.39$	$63.22 \pm 11.81$
	$\textbf{r}+\textbf{ang}$	$59.65 \pm 21.59$	$66.48 \pm 9.98$	$63.15 \pm 14.34$	$63.24 \pm 8.96$
	$\textbf{r}+\textbf{v}+\textbf{a}$	$68.98 \pm 15.15$	$67.16 \pm 14.19$	$63.89 \pm 17.76$	$66.82 \pm 9.50$
	$\textbf{r}+\textbf{v}+\textbf{ang}$	$66.20 \pm 19.74$	$68.93 \pm 13.23$	$61.76 \pm 16.73$	$65.60 \pm 9.96$
	$\textbf{r}+\textbf{a}+\textbf{ang}$	$67.04 \pm 22.66$	$70.25 \pm 16.85$	$62.78 \pm 17.72$	$66.95 \pm 12.06$
	$\textbf{r}+\textbf{v}+\textbf{a}+\textbf{ang}$	$64.81 \pm 20.21$	$64.98 \pm 13.70$	$66.57 \pm 17.34$	$65.76 \pm 10.39$
Transformer (ours)	$\textbf{r}$	$70.23 \pm 22.42$	$76.62 \pm 18.20$	$73.25 \pm 17.59$	$72.57 \pm 11.89$
	$\textbf{r}+\textbf{v}$	$74.49 \pm 22.39$	$78.56 \pm 20.08$	$71.04 \pm 16.55$	$74.61 \pm 11.94$
	$\textbf{r}+\textbf{a}$	$72.50 \pm 23.13$	$74.95 \pm 17.04$	$74.07 \pm 16.46$	$73.69 \pm 11.79$
	$\textbf{r}+\textbf{ang}$	$70.74 \pm 20.48$	$76.11 \pm 13.12$	$71.13 \pm 18.60$	$72.20 \pm 8.45$
	$\textbf{r}+\textbf{v}+\textbf{a}$	$71.76 \pm 24.54$	$72.64 \pm 20.88$	$79.31 \pm 18.02$	$\mathbf {74.95 \pm 13.77}$
	$\textbf{r}+\textbf{v}+\textbf{ang}$	$75.09 \pm 22.29$	$74.77 \pm 19.06$	$67.14 \pm 17.99$	$71.58 \pm 14.25$
	$\textbf{r}+\textbf{a}+\textbf{ang}$	$67.41 \pm 24.79$	$77.82 \pm 17.20$	$74.63 \pm 22.00$	$73.21 \pm 11.88$
	$\textbf{r}+\textbf{v}+\textbf{a}+\textbf{ang}$	$74.26 \pm 20.39$	$73.89 \pm 18.79$	$72.17 \pm 19.27$	$72.65 \pm 11.76$

Bold values indicate the best overall accuracy among each method

**Table 7:** Detailed results for three-way lowering load prediction

Method	Features	Shoulder	Knuckle	Mid-shin	All
CNN	$\textbf{r}$	$39.92 \pm 16.91$	$39.59 \pm 14.81$	$40.31 \pm 9.75$	$39.98 \pm 9.73$
	$\textbf{r}+\textbf{v}$	$49.18 \pm 17.43$	$42.54 \pm 10.62$	$44.20 \pm 10.61$	$\mathbf {44.96 \pm 7.89}$
	$\textbf{r}+\textbf{a}$	$46.68 \pm 18.56$	$42.35 \pm 8.83$	$40.80 \pm 13.58$	$43.03 \pm 7.35$
	$\textbf{r}+\textbf{ang}$	$44.26 \pm 16.80$	$45.54 \pm 10.41$	$43.06 \pm 11.26$	$43.82 \pm 8.72$
	$\textbf{r}+\textbf{v}+\textbf{a}$	$48.42 \pm 16.84$	$41.12 \pm 12.39$	$43.59 \pm 13.66$	$44.06 \pm 8.77$
	$\textbf{r}+\textbf{v}+\textbf{ang}$	$39.30 \pm 17.03$	$42.67 \pm 9.63$	$44.91 \pm 17.44$	$42.37 \pm 9.67$
	$\textbf{r}+\textbf{a}+\textbf{ang}$	$41.69 \pm 16.16$	$39.22 \pm 7.23$	$42.96 \pm 16.30$	$41.15 \pm 8.66$
	$\textbf{r}+\textbf{v}+\textbf{a}+\textbf{ang}$	$42.04 \pm 15.59$	$44.10 \pm 10.84$	$43.36 \pm 17.06$	$42.81 \pm 8.75$
LSTM	$\textbf{r}$	$42.27 \pm 11.46$	$46.33 \pm 10.82$	$46.10 \pm 14.08$	$45.19 \pm 7.22$
	$\textbf{r}+\textbf{v}$	$43.40 \pm 13.74$	$48.11 \pm 11.44$	$45.09 \pm 11.26$	$\mathbf {45.77 \pm 7.76}$
	$\textbf{r}+\textbf{a}$	$43.43 \pm 13.58$	$43.64 \pm 8.98$	$42.46 \pm 10.04$	$43.26 \pm 5.39$
	$\textbf{r}+\textbf{ang}$	$36.81 \pm 15.98$	$46.76 \pm 14.44$	$44.25 \pm 11.29$	$42.84 \pm 8.02$
	$\textbf{r}+\textbf{v}+\textbf{a}$	$41.49 \pm 12.76$	$44.81 \pm 9.99$	$45.15 \pm 11.12$	$44.28 \pm 6.77$
	$\textbf{r}+\textbf{v}+\textbf{ang}$	$39.45 \pm 12.89$	$48.08 \pm 10.90$	$44.62 \pm 12.13$	$44.21 \pm 8.61$
	$\textbf{r}+\textbf{a}+\textbf{ang}$	$37.44 \pm 15.58$	$45.20 \pm 10.08$	$44.59 \pm 10.69$	$42.65 \pm 7.25$
	$\textbf{r}+\textbf{v}+\textbf{a}+\textbf{ang}$	$42.74 \pm 17.36$	$45.28 \pm 11.24$	$44.28 \pm 12.03$	$44.25 \pm 7.95$
Transformer (ours)	$\textbf{r}$	$51.28 \pm 16.62$	$47.72 \pm 21.33$	$50.27 \pm 18.37$	$49.15 \pm 11.11$
	$\textbf{r}+\textbf{v}$	$49.06 \pm 18.01$	$50.31 \pm 17.67$	$51.47 \pm 17.64$	$49.77 \pm 10.13$
	$\textbf{r}+\textbf{a}$	$57.12 \pm 16.65$	$53.62 \pm 17.87$	$47.75 \pm 16.42$	$52.15 \pm 10.10$
	$\textbf{r}+\textbf{ang}$	$45.13 \pm 17.98$	$50.34 \pm 15.07$	$52.07 \pm 15.88$	$49.16 \pm 7.06$
	$\textbf{r}+\textbf{v}+\textbf{a}$	$53.96 \pm 14.72$	$51.51 \pm 18.43$	$52.50 \pm 16.59$	$\mathbf {52.26 \pm 9.54}$
	$\textbf{r}+\textbf{v}+\textbf{ang}$	$49.15 \pm 20.29$	$53.03 \pm 20.94$	$47.65 \pm 17.07$	$50.30 \pm 7.44$
	$\textbf{r}+\textbf{a}+\textbf{ang}$	$52.67 \pm 17.93$	$49.34 \pm 19.75$	$54.43 \pm 16.39$	$51.37 \pm 9.72$
	$\textbf{r}+\textbf{v}+\textbf{a}+\textbf{ang}$	$48.46 \pm 14.14$	$54.99 \pm 17.59$	$45.92 \pm 14.73$	$49.40 \pm 7.43$

Bold values indicate the best overall accuracy among each method

### Ablation studies

We conduct ablation studies to investigate the design of our model, and the effects of input kinematic features. This is done by varying number of Transformer blocks in our design and considering different combinations. Additionally, we evaluate an intra-subjects setting, where a model is trained and evaluated on disjoint sets of data from the same participants (e.g., different loads from the same subject). Detailed results are presented in Tables 2, 3, 4, 5, 6, 7. We provide further discussions below.

*Model design*. We evaluate the effects of the number of Transformer blocks, from shallow to deep ($L \in \{2,4,6\}$) in Table 2. Increasing the number of blocks generally results in minor improvements in mean accuracy, albeit with a slight increase in variance. For example, our model with $L = 6$ achieves $53.17\% \pm 11.12\%$ accuracy in the three-way classification of lowering load, compared to $49.40\% \pm 7.43\%$ accuracy by its shallower version ($L = 2$). These results suggest that further optimization of model architecture, potentially with the help of a larger-scale dataset, may lead to additional performance improvements.

*Effects of kinematic features*. Our experiment begins with joint positions *r*, and gradually add different combinations of kinematics features including velocity *v*, acceleration *a*, and joint angles *ang*. Using the combination of *r*, *v*, *a* yields the best overall performance of our model. In contrast, including *ang* tends to improve accuracy for one pose type at the cost of degrading performance for the other, ultimately reducing overall accuracy. Notably, we observe that using body joint positions *r* plus velocity *v* or acceleration *a* leads to competitive and sometimes slightly better accuracy, especially for simpler binary lifting load prediction. We conjecture that this is likely due to interdependency among the kinematic features: our Transformer might learn to compute additional features within the model itself.

We also notice that our baselines (CNN and LSTM) do not significantly benefit from using the full set of kinematic features. They often achieve their highest accuracy using only joint positions using joint positions *r* or with the addition of velocity *v* or acceleration *a* features.

*Intra-subject versus inter-subject generalization*. Our main results are obtained using leave-one-subject-out cross-validation, which evaluates model generalization across subjects (inter-subject). To further examine model performance, we also conduct an additional experiment that assesses generalization within subjects (intra-subject), and compare the results against the inter-subject setting. This experiment follows an intra-subject split strategy. Specifically, for each subject, a subset of samples from each lifting load category at every posture is held out for testing, while the remaining samples are used for training. Multiple folds are then generated by randomly varying the held-out samples across individuals.

Table 3 presents results of the intra-subject setting with 3-fold cross-validation, and compares them against the inter-subject setting. In the inter-subject split, the training set averages about $94\%$ of the data, whereas in the intra-subject split it’s about $65\%$. In comparison to the inter-subject setup, the intra-subject setting has smaller training sets and substantially larger test sets, yet it achieves similar or slightly higher accuracies across tasks. We conjecture that, within the same subject, motion trajectories corresponding to a given lifting load exhibit consistent patterns, which may provide more reliable cues for quantifying loads, although our current data do not allow us to directly test this hypothesis.

## Conclusion and discussion

In this paper, we presented a method for estimating lifting and lowering load from input video, with the goal of enabling video-based injury risk assessment for manual material handling tasks. Our approach detects and tracks key body parts (i.e., joints), extracts kinematic features, and learns a Transformer-based model to predict lifting and lowering load levels. Based on a controlled laboratory study, our experiments demonstrate that the proposed method achieves significantly higher accuracy in estimating both lifting and lowering loads compared to baseline models using CNNs and LSTMs. Grounded in the broader challenge of intuitive physics, our work represents an initial step toward non-invasive and scalable video-based tools for injury risk assessment.

*Limitation*. While our work demonstrates the feasibility of video-based load estimation, several limitations should be acknowledged. First, our current model estimates lifting load categories (e.g., light, medium, heavy) rather than the exact load, and therefore cannot yet be directly applied in the RNLE. Nonetheless, it is important to note that the RNLE evaluates injury risk by comparing the calculated lifting index against a fixed threshold: any value above 1.0 indicates increased risk. This implies that even approximate load estimates may still provide meaningful information for quantifying injury risk. Second, the study focuses solely on symmetric lifting from sagittal views in a controlled laboratory setting, which captures important but not all real-world scenarios. In practice, lifting often involves more complex and asymmetric movements. Third, our method considers absolute weight of lifting loads, without accounting for individual differences in strength and exertion. In reality, the same weight may correspond to different levels of physical effort across participants. Finally, this study is based on a relatively small sample size (19 participants), which may limit the generalizability of some of our findings.

*Future directions*. Future work should shift from discrete load categories to continuous load estimation, extend to asymmetric lifts observed from diverse viewpoints, and incorporate measures of individual strength. Continuous load prediction would help close the gap with existing ergonomics risk assessment tools. Accounting for asymmetric lifts and diverse viewpoints would better capture the variability of real-world scenarios. Incorporating individual strength would enable estimation of relative load as a percentage of maximum capacity. This opens the opportunity to develop new lifting ergonomics tools that do not require the exact load. Pursuing these directions will likely require learning from large-scale datasets. While large-scale training has achieved remarkable success in many computer vision problems [47], it presents significant privacy concerns in the context of injury risk assessment. One promising direction is to investigate privacy-preserving learning techniques such as differential privacy [48] and federated learning [49].

Another avenue for future research is the use of multi-camera setups to capture body motion from multiple viewing angles [50], enabling more accurate 3D trajectory estimation for lifting load prediction. However, deploying such systems in real-world workplaces poses practical challenges, particularly in environments where there is limited flexibility for multiple cameras. A parallel direction is to consider monocular 3D pose estimation methods [51, 52], capable of recovering 3D joint locations from a single camera. Although their application to ergonomics risk assessment remains an open question, recent evidence suggests that their accuracy is sensitive to camera viewpoint, with the sagittal view used in our study producing the lowest error. [53]. With access to 3D pose data, more advanced data augmentation strategies could be explored beyond simple techniques such as random flipping or Gaussian noise. For example, combining parametric human body models [23] with biomechanics simulation engines [54] would allow the synthesis of anthropometric variability and the generation of diverse yet biomechanically realistic training samples, albeit at the cost of substantial computational resources.
